# Bioinformatic and *in vitro* Analyses of Arabidopsis Starch Synthase 2 Reveal Post-translational Regulatory Mechanisms

**DOI:** 10.3389/fpls.2018.01338

**Published:** 2018-09-19

**Authors:** Jenelle A. Patterson, Ian J. Tetlow, Michael J. Emes

**Affiliations:** Department of Molecular and Cellular Biology, University of Guelph, Guelph, ON, Canada

**Keywords:** *Arabidopsis thaliana*, starch biosynthesis, starch synthase 2, post-translational regulation, protein phosphorylation, protein-protein interactions, oligomerization, casein kinase II

## Abstract

Starch synthase 2 (SS2) is an important enzyme in leaf starch synthesis, elongating intermediate-length glucan chains. Loss of SS2 results in a distorted starch granule phenotype and altered physiochemical properties, highlighting its importance in starch biosynthesis, however, the post-translational regulation of SS2 is poorly understood. In this study, a combination of bioinformatic and *in vitro* analysis of recombinant SS2 was used to identify and characterize SS2 post-translational regulatory mechanisms. The SS2 N-terminal region, comprising the first 185 amino acids of the mature protein sequence, was shown to be highly variable between species, and was predicted to be intrinsically disordered. Intrinsic disorder in proteins is often correlated with protein phosphorylation and protein-protein interactions. Recombinant *Arabidopsis thaliana* SS2 formed homodimers that required the N-terminal region, but N-terminal peptides could not form stable homodimers alone. Recombinant SS2 was shown to be phosphorylated by chloroplast protein kinases and recombinant casein kinase II at two N-terminal serine residues (S63, S65), but mutation of these phosphorylation sites (Ser>Ala) revealed that they are not required for homo-dimerization. Heteromeric enzyme complex (HEC) formation between SS2 and SBE2.2 was shown to be ATP-dependent. However, SS2 homo-dimerization and protein phosphorylation are not required for its interaction with SBE2.2, as truncation of the SS2 N-terminus did not disrupt ATP-dependent HEC assembly. SS2 phosphorylation had no affect on its catalytic activity. Intriguingly, the removal of the N-terminal region of SS2 resulted in a 47-fold increase in its activity. As N-terminal truncation disrupted dimerization, this suggests that SS2 is more active when monomeric, and that transitions between oligomeric state may be a mechanism for SS2 regulation.

## Introduction

Starch is a complex glucan polymer made by plants as an osmotically inert carbohydrate storage molecule, which is exploited for human nutrition and as a binding agent in many industrial processes (Tetlow et al., 2004a; Tetlow, 2006). Starch is typically produced either as a temporary storage compound, such as in leaf chloroplasts during the day to be degraded at night to support respiration, or longer-term, such as in endosperm, to support germination of the next generation. Starch exists as insoluble, semi-crystalline granules composed of two glucose-polymers, amylose and amylopectin, which contain linear α-(1,4) glycosyl units that are branched via α-(1,6) linkages (Badenhuizen, 1963). Amylopectin is highly branched, comprises 70–90% of the starch granule (Deatherage et al., 1955), and its composition determines granule crystallinity. Amylopectin is synthesized through the coordinated activities of starch synthases (SS), which elongate linear chains; starch branching enzymes (SBE), which sequentially cleave α-(1,4) bonds and add branch points via α-(1,6) linkages; and starch debrancing enzymes (DBE), which trim excess branch points to maintain amylopectin compaction and crystallinity (Martin and Smith, 1995; Mouille et al., 1996). In most plant species, multiple isozymes exist within each enzyme class, and their number, localization, substrate affinity and expression vary depending on plant species, tissue-type and developmental stage.

Starch synthase isoforms differ with respect to the preferred glucan chain length of their substrates. SS2 elongates intermediate length glucan chains (DP 12-25), extending the short chains produced by SS1 (Commuri and Keeling, 2001; Morell et al., 2003; Kosar-Hashemi et al., 2007; Zhang et al., 2008; Cuesta-Seijo et al., 2016). SS2 is a key enzyme in starch biosynthesis, as evident by the phenotype caused by SS2 null mutations. Starch from *ss2*- plants exhibits increased apparent amylose content; altered amylopectin chain-length distribution (CLD), with increased short chains (DP 4-7) and decreased intermediate chains (DP 11-19); lower gelatinization temperature; distorted granule morphology; and altered granule crystallinity and viscosity (Li and Corke, 1999; Perera et al., 2001; Zhang et al., 2004). Starch produced by *ss2*- mutants is more suitable for industrial applications due to its altered physiochemical properties, including lower gelatinization temperature, lower viscosity, faster rate of hydrolysis, and increased stability of gel hardness and adhesiveness over time and after freeze-thaw storage (Li and Corke, 1999; Perera et al., 2001; Zhang et al., 2004). In rice, mutations in SS2 in the endosperm result in starches with favorable cooking properties which are exploited in *japonica* rice lines (Nakamura et al., 2005). SS2 also forms the core of a functional heteromeric enzyme complex (HEC) involving other SS isoforms and SBE, which has been demonstrated in wheat, barley, maize and rice (Tetlow et al., 2004b, 2008; Hennen-Bierwagen et al., 2008; Liu et al., 2009, 2012a; Ahmed et al., 2015; Crofts et al., 2015), as well as Arabidopsis (Zhao, unpublished). This HEC is active at the granule surface, but becomes entrapped within the granule during amylopectin synthesis. Loss or mutation of SS2 affecting its ability to bind amylopectin decreases or abolishes granule association of other HEC enzymes, indicating that SS2 plays a critical role in HEC granule-association (Grimaud et al., 2008; Liu et al., 2012b; Luo et al., 2015).

Assembly of the aforementioned SS-SBE HECs in maize, wheat and barley endosperm is phosphorylation-dependent (Tetlow et al., 2004b, 2008; Ahmed et al., 2015). Furthermore, independent of enzyme complex association, wheat SBEIIa and SBEIIb, and maize SBEIIb show reduced catalytic activity following protein dephosphorylation (Tetlow et al., 2004b). Although SS2 phosphopeptides have been detected in wheat, maize, barley, and Arabidopsis (Tetlow et al., 2004b, 2008; Reiland et al., 2009; Meyer et al., 2012; Ahmed et al., 2015; Chen et al., 2016), the role of SS2 phosphorylation in HEC assembly and any effect on catalytic activity has yet to be determined.

The majority of SS2 characterization has historically been performed by mutational analysis of commercially significant endosperm isoforms, particularly in barley (Morell et al., 2003), rice (Umemoto et al., 2002; Nakamura et al., 2005), wheat (Yamamori et al., 2000; Tetlow et al., 2008), and maize (Hennen-Bierwagen et al., 2008; Liu et al., 2012b), though there are some studies of SS2 in pea embryo (Denyer and Smith, 1992; Craig et al., 1998) and, more recently, Arabidopsis leaves (Zhang et al., 2008; Szydlowski et al., 2011; Pfister et al., 2014). However, little is currently known about the mechanisms that regulate leaf SS2 isoforms, despite leaf starch playing a key role in source-sink relationship in plants.

Identifying the mechanisms that control starch biosynthetic enzymes *in planta* is complicated by the interconnectedness of the regulatory mechanisms themselves, and by functional redundancy among isoforms. For enzymes that belong to characterized and evolutionarily-conserved pathways, information concerning protein structure, mode of action, active sites, and post-translational regulatory mechanisms can be exploited. In this study, a systematic approach combining bioinformatics and *in vitro* analyses was used to identify mechanisms by which SS2 could be regulated, including protein phosphorylation and through protein-protein interactions.

## Materials and methods

### Bioinformatics analysis

To analyse sequence identity, amino acids sequences supplied by the National Center for Biotechnology Information (NCBI; www.ncbi.nlm.nih.gov) were aligned using PRALINE multiple sequence alignment (MSA; www.ibi.vu.nl/programs/pralinewww/). Clustal Omega (www.ebi.ac.uk/Tools/msa/clustalo/) was used to quantify % sequence identity. Arabidopsis SS2 sequences were analyzed for intrinsically disordered regions (IDR) using Predictor of Natural Disordered Regions (PONDR; www.pondr.com/). Putative protein phosphorylation sites were predicted using KinasePhos (http://kinasephos.mbc.nctu.edu.tw/). AtSS2 structural models were produced using I-TASSER (Iterative Threading ASSEmbly Refinement; https://zhanglab.ccmb.med.umich.edu/I-TASSER/) with default settings and no additional restraints. Additional crystal structures were accessed using the RCSB PDB Protein Data Base (www.rcsb.org/). Homology models and crystal structures were analyzed using PyMOL 1.2r1 (www.pymol.org).

### Cloning, expression and purification of recombinant arabidopsis SS2

Mature Arabidopsis SS2 cDNA (amino acids 55-792 referring to its full-length sequence, Genbank accession number AHL38788.1) was amplified from rosette leaf total mRNA, and cloned into a binary vector pET29a (Novagen catalog number 69871-3), adding an N-terminal S-tag. SS2 cDNA was mutated using PCR site directed mutagenesis (SDM), based on the protocol by QuikChange site-directed mutagenesis kit (Stratagene, catalog number 200518). Truncated SS2 cDNAs were produced by adding KPN1 restriction enzyme cut sites (underlined) at 171bp, 390bp, 522bp, and 708bp of the SS2 cDNA sequence. The pET29a vector contains a KPN1 cut site 27bp before the beginning of the SS2 cDNA sequence, following the S-tag coding sequence. The respective forward primer sequences were: 5′- GCA CAC CTG ACT TG*G GTA CC*G CAA AAG CTT CTT CC−3′, 5′- GCC TCT GTG ATA TCT *GGT ACC* CCT GTA ACC TCT CC−3′, 5′- CTC CTG AGA AAA CT*G GTA CC*C CTG TAA CTT CTC C−3′, 5′- GGC AAG GAT GAA GAG AAG *GGT ACC* CCA CTG GCT GGA GC−3′. Reverse complements of each primer were used as reverse primers. Digesting the resultant PCR fragments with KPN1 and re-ligating the sequences produced polypeptides truncated from the N-terminus as follows: 57-792 (TRU1), 104-792 (TRU3), 176-792 (TRU4), and 240-792 (TRU5). A polypeptide covering just the N-terminal region, 1-240 (NT), was produced by adding a stop codon (underlined) at 708 bp using the forward primer 5′- GGC AAG GAT GAA GAG AAC *TAG* TCT CCA CTG GCT GG−3′, and its reverse compliment to amplify the SS2 cDNA sequence. PCR SDM was used to generate Ser>Ala mutations at putative phosphorylation sites identified by Reiland et al. (2009), Ser63 and Ser65. Primers used for SS2 PCR SDM's were as follows: forward, 5′- GCG TCG AGG CT*G CT*G GCT CAG ACG−3′, reverse, 5′- CGT CTG AGC C*AG C*AG CCT CGA CGC−3′ for Ser63>Ala; forward, 5′- GAG GCT TCC GGC *GCA* GAC GAT GAT GAG CC−3′, reverse, 5′- GGC TCA TCA TCG TC*T GC*G CCG GAA GCC TC−3′ for Ser65>Ala; and forward, 5′- GTC GAG GCT *GCT* GGT *GCT* GAC GAT GAT GAG−3′, reverse, 5′- CTC ATC ATC GTC *AGC* ACC *AGC* AGC CTC GAC−3′ for Ser63/65>Ala (mutated nucleotides for Ala are underlined). PCR reactions were performed using iProof High-fidelity DNA Polymerase kit (Biorad Cat No. 172-5331), with 25 μl reactions containing: 5 μl of 10x GC buffer, 100 ng of vector, 100 ng of each forward and reverse primers, 1 μl of 10 mM MgCl_2_, 1 μl of DMSO, and 1 μl of 10 mM dNTP mixture (Invitrogen). Cycling parameters were: initial denaturation of 98°C for 30 s; 18 cycles of 98°C for 15 s, 45–55°C for 30 s, 72°C for 2 min; and a final extension at 72°C for 5 min. Following cycling, Dam-methylated parental DNA was digested using FastDigest Dpn1. Five microlitres of the digested SDM PCR product was transformed into 50ul of competent *E. coli* DH5α, and transformants plated on LB- agar plates containing 50 μg/ml kanamycin. DNA was isolated from resulting colonies and sequenced to confirm the mutated sites. Recombinant plasmids were transformed into ArcticExpress competent cells (Stratagene Cat. No. 230193) and proteins were expressed by inducing with 1 mM isopropyl-D-thiogalactopyranoside (IPTG) at 10°C, 250 rpm for 24 h. ArcticExpress cells were collected by centrifugation and lysed using Biobasic's “Extract-EZ B, Bacterial Protein Extraction Kit” (Cat No. BS596), modified by using Native Purification Buffer (50 mM NaH_2_PO_4_, pH 8.0; 0.5 M NaCl) in lieu of the provided buffer.

### Plant material and plastid extract isolation

Wild type *Arabidopsis thaliana* ecotype Columbia was grown in the University of Guelph phytotron under a regime of 16 h light/8 h dark, 22°C/18°C, ambient humidity, 150 μmol photons m^−2^ s^−1^ light intensity in “L4 Sunshine Mix” soil for 22 days. Chloroplast extracts were prepared by harvesting mature rosette leaf tissue 1 h before light (end of night), and submerging immediately in pre-cooled grinding buffer (50 mM HEPES-KOH, pH 7.5; 330 mM sorbitol; 1 mM MgCl_2_; 1 mM MnCl_2_), on ice. Leaves were homogenized and filtered through Miracloth (CalBiochem Cat No. 475855), before centrifuging at 1,000 g at 4°C for 8 min. The chloroplast-enriched pellet was resuspended and lysed in rupturing buffer (RB) (20 mM Tricine, pH 7.5; 7.5 mM MgCl_2_; 1 mM DTT and 1x ProteaseArrest protease inhibitor cocktail [G-Biosciences Cat No. 786-332)]. To remove starch and membranes, lysed chloroplasts were centrifuged at 14,000 g for 10 min at 4°C, and the supernatant flash-frozen in liquid nitrogen and stored at −80°C until needed. Maize endosperm amyloplasts were prepared from fresh kernels ~22 days after pollination using a modified method previously described by Tetlow et al. (2008). The plastids were osmotically lysed with rupturing buffer, described above.

### Phosphorylation of recombinant SS2

Wild type and mutated, recombinant S-tagged SS2 proteins were phosphorylated with plastid extracts or recombinant casein kinase II (CKII) (NEB Cat No. P6010S) using radiolabeled [γ-^32^P]-ATP. 1 ml lysate from ArcticExpress bacteria expressing S-tagged SS2 (1 mg/ml) was incubated with (0.05 ml) S-protein agarose beads (Sigma Aldrich Cat. No. 69704) overnight at 4°C and equilibrated in RB prior to phosphorylation. When using plastid extracts as a source of protein kinase, the reaction mixture contained: 100 μM ATP; 1 μC [γ-^32^P]-ATP; 0.5 mM CaCl_2_; 1x ProteaseArrest; 1 mM DTT; 25 μl SS2 immobilized to S-tag beads; and 960 μl chloroplast stroma (~1 mg/ml total protein) in a 1 ml total reaction volume. When using recombinant CKII, the reaction mixture contained: 100 μM ATP; 1 uC [γ-^32^P]-ATP; 0.5 mM CaCl_2_; 1x ProteaseArrest; 1 mM DTT; 2 μl SS2 immobilized to S-tag beads; 100 U CKII; and 210 μl RB in a total reaction volume of 250 μl, incubated at room temperature for 20 min with rotation. S-protein agarose beads were washed three times by adding 1x S-tag Wash Buffer (20 mM Tris-HCl, pH 7.5; 150 mM NaCl; 0.1% Triton-X100), before boiling in SDS-loading dye and loading onto 10% (w/v) SDS-gels. Separated proteins were transferred to nitrocellulose membranes, and stained with Ponceau-S. ^32^P-labeled proteins were detected by autoradiography with BioMax light film (KODAK Cat No. MKBR6926).

### Size-exclusion chromatography

Wild type and mutated, recombinant SS2 protein was fractionated using gel permeation chromatography (GPC) to analyze oligomerization state on a Superdex 20/300 GL column (Amersham Pharmacia Biotech, USA) connected to an AKTA fast protein liquid chromatography (FPLC) system (Amersham Biosciences), controlled using UNICORN manager software (GE Healthcare Cat No. 29-0187-51). ArcticExpress lysates containing SS2 recombinant protein were loaded onto Superdex 20/300 GL column pre-equilibrated with RB, and run with RB at a flow rate of 0.25 ml/min, collecting 0.5 ml fractions. Bacterial lysate and GPC elution fractions were boiled in SDS-loading dye, separated on 10% SDS-gels, and immunoblotted using anti-S-tag primary antibodies (Sigma-Aldrich Cat. No. SAB2702204). Wild type recombinant SS2 lysates were pretreated using phosphorylation or dephosphorylation conditions before separating on GPC by adding 2 U recombinant casein kinase II (NEB Cat No. P6010S) and 1 mM ATP, or 10U calf intestinal phosphatase (CIP)(NEB Cat No. M0290S) to 1 ml bacterial lysate, respectively, for 20 min at room temperature.

### Protein-protein interaction assay

Bacterial lysate containing recombinant SS2 protein was incubated with S-tag beads overnight at 4°C, after which the beads were equilibrated in RB. Reaction mixtures included: 0.5 mM CaCl_2_; 1x ProteaseArrest; 1 mM DTT; 25 μl SS2-S-tag beads; and 960 μl plastid stroma in a 1 ml total reaction volume. Phosphorylation and dephosphorylation reactions were performed by adding 1 mM ATP or 100U CIP, respectively, to the reaction mixtures, which were incubated at room temperature for 40 min. The S-tag immobilized SS2 and its associated proteins were washed three times by adding 1x S-tag Wash Buffer (20 mM Tris-HCl, pH 7.5; 150 mM NaCl; 0.1% Triton-X100). Washed beads were boiled in SDS-loading dye, and loaded onto 10% (w/v) SDS-gels for separation and immunoblotting with S-tag and SBE-specific primary antibodies [prepared as described by Liu et al. (2012a)].

### SS2 activity assay

Immobilized SS2 bound to S-tag beads was pre-equilibrated in 50 mM Bicine, pH 8.5. Reactions mixtures contained: 50 mM Bicine, pH 8.5; 0.7 mM phosphoenolpyruvate (PEP); 25 mM potassium acetate, pH 8.0; 0.1% (w/v) BSA; 2 mM MgCl_2_; 10 mM DTT; 4 U/ml pyruvate kinase (PK); 6 U/ml lactate dehydrogenase (LDH); 1 mM ADP-glc; 0.375 mM NADH; and 1 mg/ml corn starch (boiled in water at 95°C for 20 min, then cooled). Nine hundred and thirty-five microliter reaction mix was added to Fifty microliter equilibrated SS2 bound to S-tag beads, or to fifty microliter Bicine, pH 8.5. Reactions were started by addition of ADP-glc. Readings were taken every 15 min for 75 min, and the change in optical density at 340 nm determined over time. At each time point the reactions were centrifuged at 2,000 g for 15 s to collect supernatant, and optical density determined.

## Results

### The SS2 N-terminal region is not conserved among plant species

**Figure 1:**
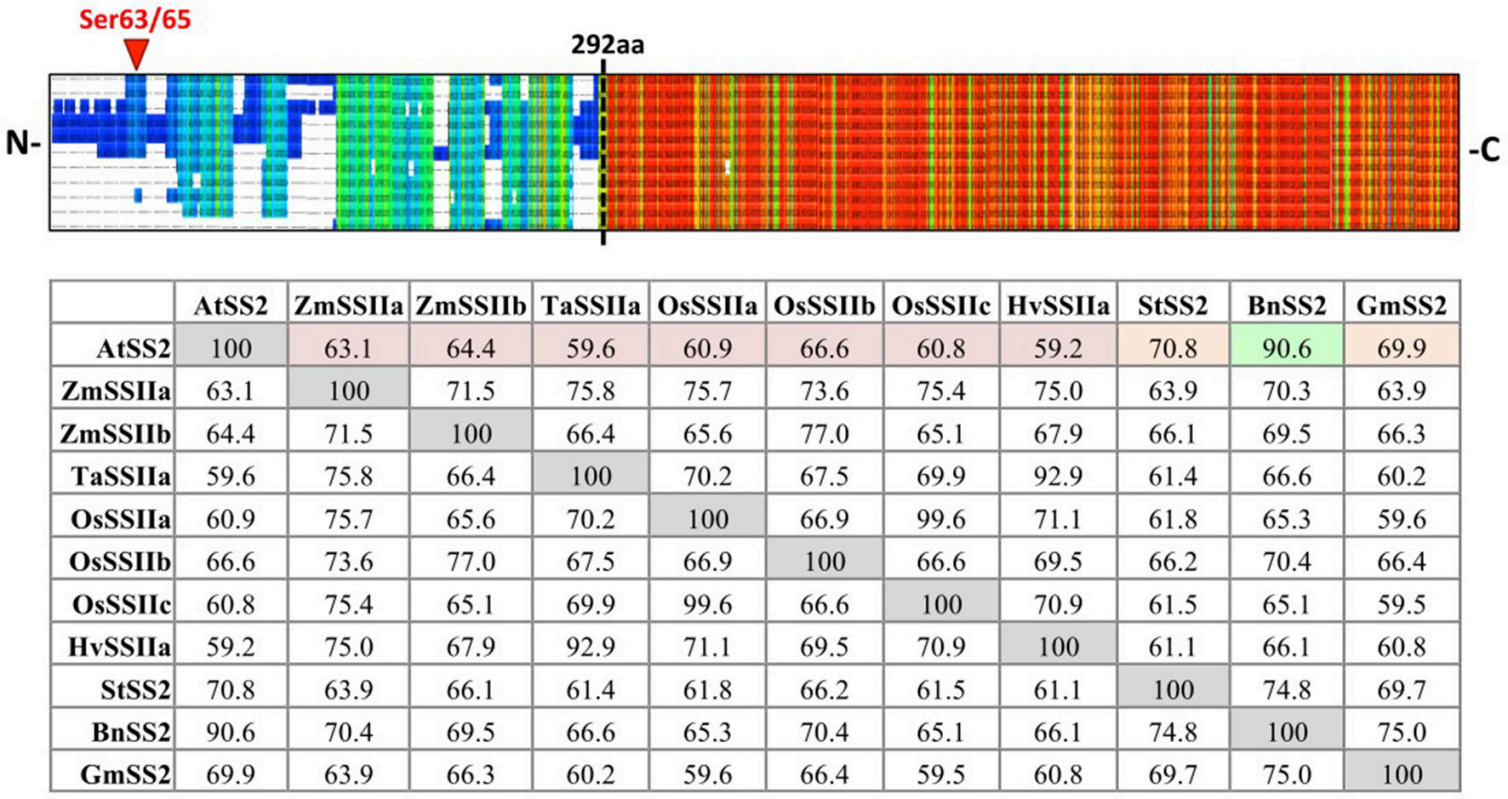
PRALINE multiple sequence alignment and Clustal Omega sequence identity (%) matrix of Arabidopsis starch synthase 2 and SS2 orthologs. Sequences aligned in descending order: *Arabidopsis thaliana* SS2 (AtSS2), *Glycine max* SS2 (GmSS2), *Triticum aestivum* SSIIa (TaSSIIa), *Oryza sativum* cv *indica* SSIIa (OsSSIIa), *Oryza sativum* cv *indica* SSIIc (OsSSIIc), *Hordeum vulgare* SSIIa (HvSSIIa), *Zea mays* SSIIa (ZmSSIIa), *Zea mays* SSIIb (ZmSSIIb), *Oryza sativum* cv *indica* SSIIb (OsSSIIb), *Solanum tuberosum* SS2 (StSS2), *Brassica napus* SS2 (BnSS2). Cool colors represent low conservation while warm colors represent high conservation. The SS2 N- termini (prior to the dashed black line) are highly variable, though the C-termini are relatively more conserved. AtSS2 shares highest sequence identity with its close evolutionary relative, BnSS2 (91%, highlighted in green), with all remaining SS2 orthologs sharing between 59 and 71% identity.

SS2 isoform sequences were aligned using PRALINE MSA (Figure 1). The N-terminal regions (NTR) share little identity between species until AtSS2 L291 (PPPLAG), even when comparing within monocotyledonous or dicotyledonous isoforms. Conversely, the sequences C-terminal to AtSS2 L291 were highly conserved among all species. Quantifying % sequence identity revealed that AtSS2 shares highest sequence identity with its closest evolutionary relative, BnSS2 (91%), with all remaining SS2 orthologs (both monocotyledonous and dicotyledonous) sharing between 59 and 71% identity to AtSS2.

### The N-terminal domain of SS2 is predicted to be intrinsically disordered

**Figure 2:**
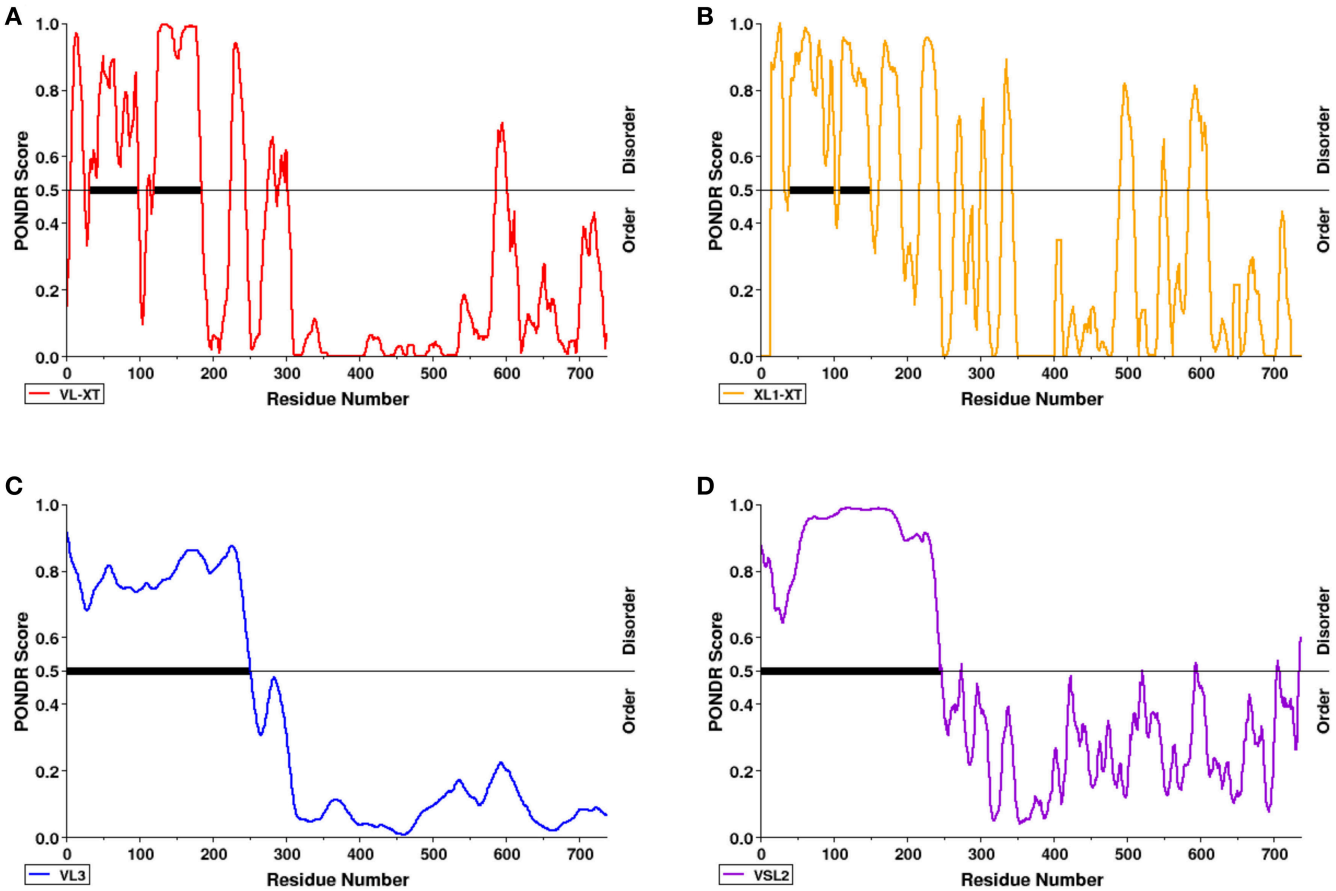
The N-terminal region of AtSS2 is predicted to be intrinsically disordered. PONDR VL-XT (red), XL1-XT (yellow), VL3 (blue), VSL2 (purple) algorithms **(A–D** in diagram) were used to analyze mature AtSS2 amino acid sequence. VL-XT and XL1-XT outputs predict two disordered regions between 50 and 100 aa and 100–200aa; VL3 and VSL2 outputs predict the entire N-terminal region (1 to ~250 aa) to be intrinsically disordered.

PONDR VL-XT, XL1-XT, VL3, and VSL2 algorithms were used to predict intrinsic disorder (unstructured coils that fluctuate between different conformations) within the AtSS2 mature sequence length (737aa total). Each algorithm output values ranged between 0 and 1, with residues exceeding a 0.5 threshold considered to be disordered (Figure 2). VL-XT and XL1-XT outputs predicted two disordered regions between 50 and 100 aa and 100–200 aa, while VL3 and VSL2 outputs predicted the entire N-terminal region (1 to ~250 aa) to be intrinsically disordered. In all cases, the majority of the AtSS2 N-terminus was predicted to be intrinsically disordered.

### Predicted structural similarity between SS2 and ScGP

**Figure 3:**
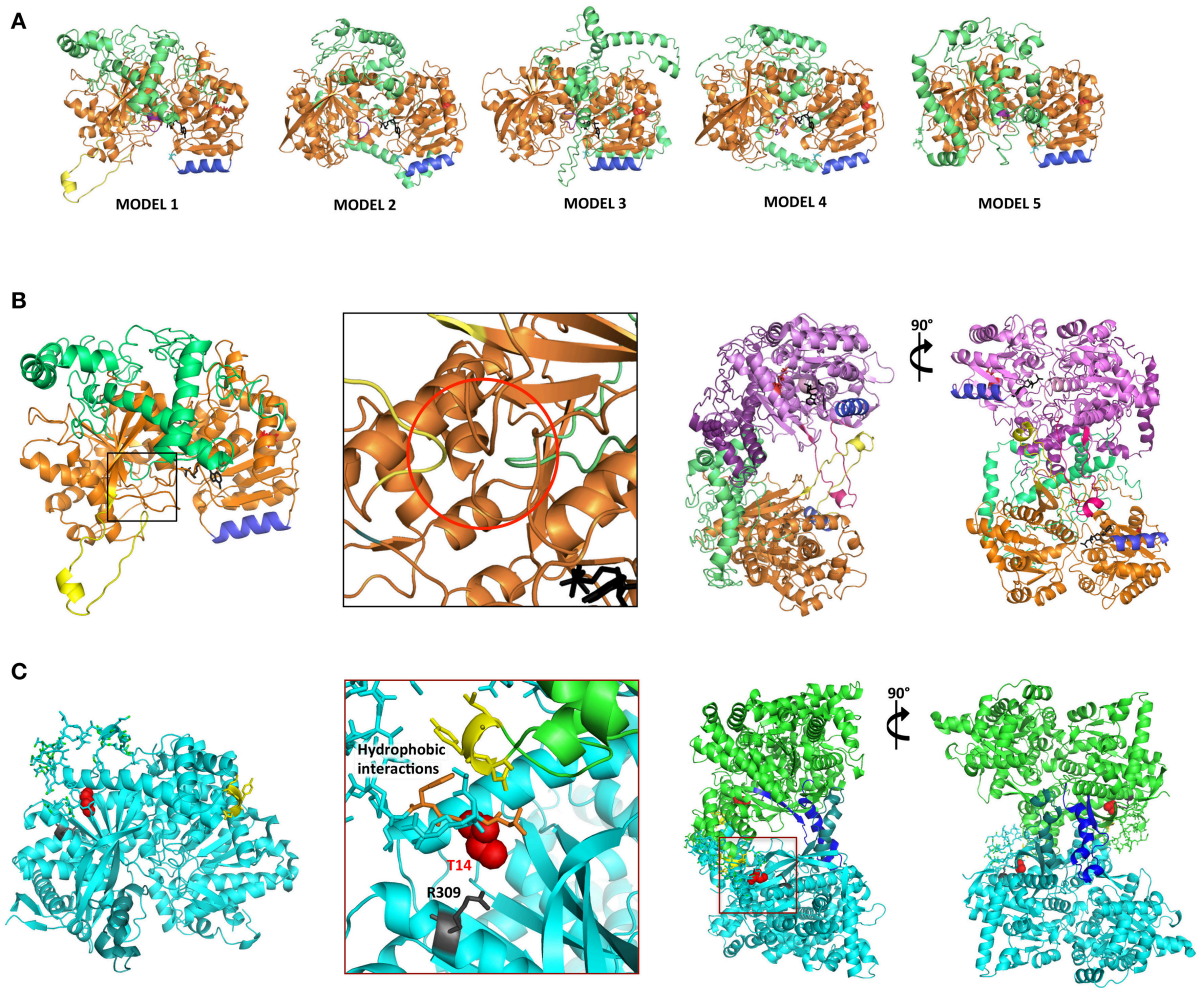
I-TASSER AtSS2 model 1 shares structural identity with Saccharomyces cerevisiae glycogen phosphorylase (ScGP). **(A)** I-TASSER AtSS2 models 1-5 color-coding: conserved central-C-terminal domains (orange and pink), disordered N-terminus 1-238 aa (green and purple), conserved coiled-coil (blue), ADP-glc (black), and model 1-specific extension (yellow). **(B)** Specific to I-TASSER AtSS2model 1, an N-terminal loop (green) is threaded within the C-terminal catalytic region (orange), which forces the protrusion of the central loop (yellow) (red circle). I-TASSER AtSS2 model 1 (orange/pink) was aligned to ScGP homodimer and revealed that the model 1-specific extension (yellow/hot pink) is conserved to ScGP alpha-helical extension. The disordered AtSS2 N-terminus 1–238aa (green/purple) may stabilize homodimerization similar to ScGP CAP region. **(C)** ScGP (PDB ID: 1YGP; cyan and green) homodimerization interface is formed by its alpha-helical extension (teal and blue). N-terminal phosphothreonine 14 (red spheres) interacts with R309 (gray), allowing interactions of hydrophobic N-terminus (orange) with “CAP” region of binding partner (yellow sticks) (Lin et al., 1996).

Three-dimensional homology modeling of mature AtSS2 was used to analyze sites of interest predicted from amino acid sequence, and to compare with structurally similar protein analogs. Using I-TASSER, five AtSS2 structural models were produced using iterative template fragment assembly from multiple threading templates (Figure 3A). The conserved, catalytic C-terminal region of the AtSS2 I-TASSER models were similar amongst models 2–5. The model 1 C-terminus differed from models 2–5 by the inclusion of a central alpha-helical/loop extension (Figure 3B), which was caused by the threading of an N-terminal loop through the catalytic cleft. I-TASSER identified *Saccharomyces cerevisiae* glycogen phosphorylase (ScGP, PDB ID: 1YGP) as a structural analog to AtSS2 model 1 (Figures 3B,C). The ScGP homodimerization interface was formed by alpha-helical extension, and was stabilized by N-terminal interactions. The N-terminal phosphothreonine 14 interacts with R309, allowing interactions of hydrophobic N-terminus with “CAP” region of the binding partner (Lin et al., 1996). Aligning the I-TASSER SS2 model 1 structures to ScGP homodimers revealed that SS2 may homodimerize in a similar manner to ScGP.

### SS2 phosphorylation by chloroplast protein kinases and recombinant CKII

**Figure 4:**
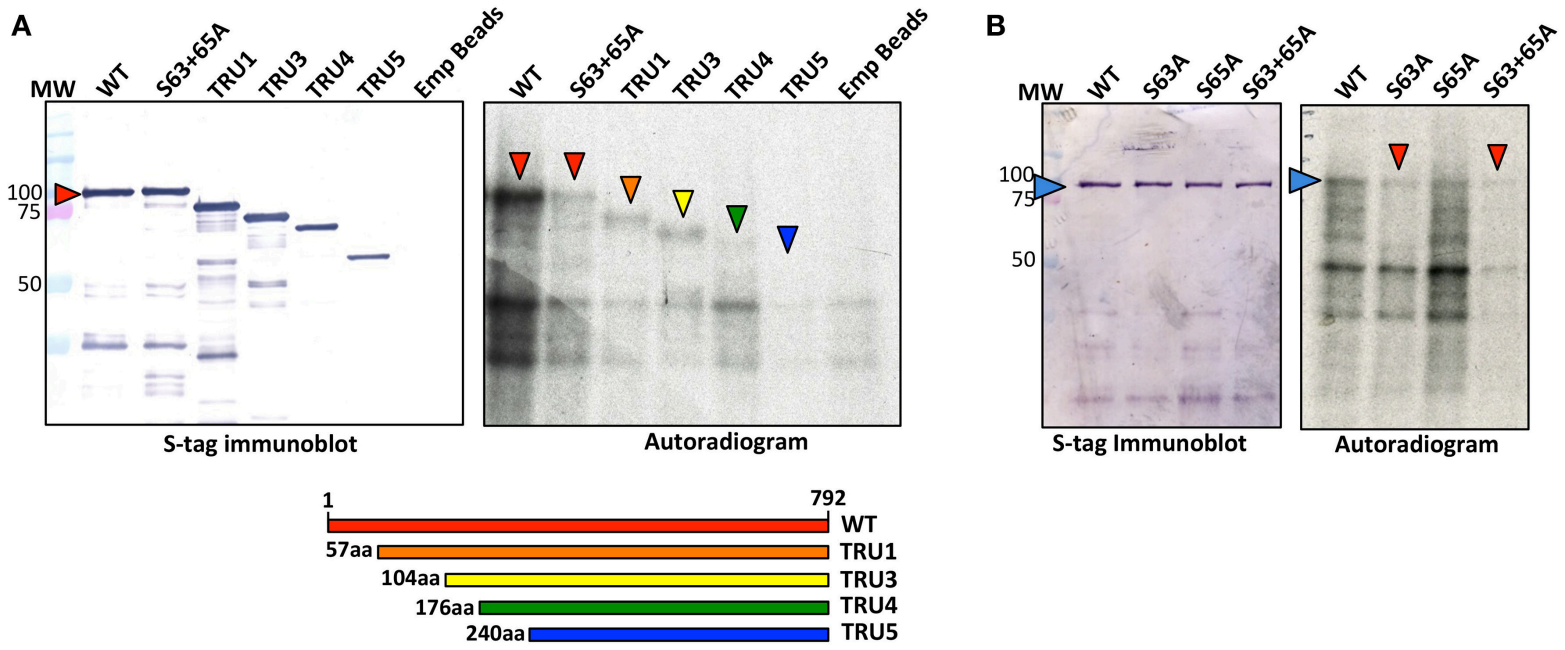
Phosphorylation of recombinant wild type and mutated SS2. S-tag agarose immobilized recombinant SS2 proteins containing N-terminal truncations **(A)** or point mutations **(B)** were incubated with [γ-^32^P]-ATP and chloroplast extracts. SS2 proteins (arrowheads) were separated on 10% SDS-gels, transferred to a nitrocellulose membrane and immunoblotted with SS2 or S-tag primary antibodies (left), and ^32^P-phosphorylation visualized by autoradiography (right). MW = molecular weight markers (kDa).

**Figure 5:**
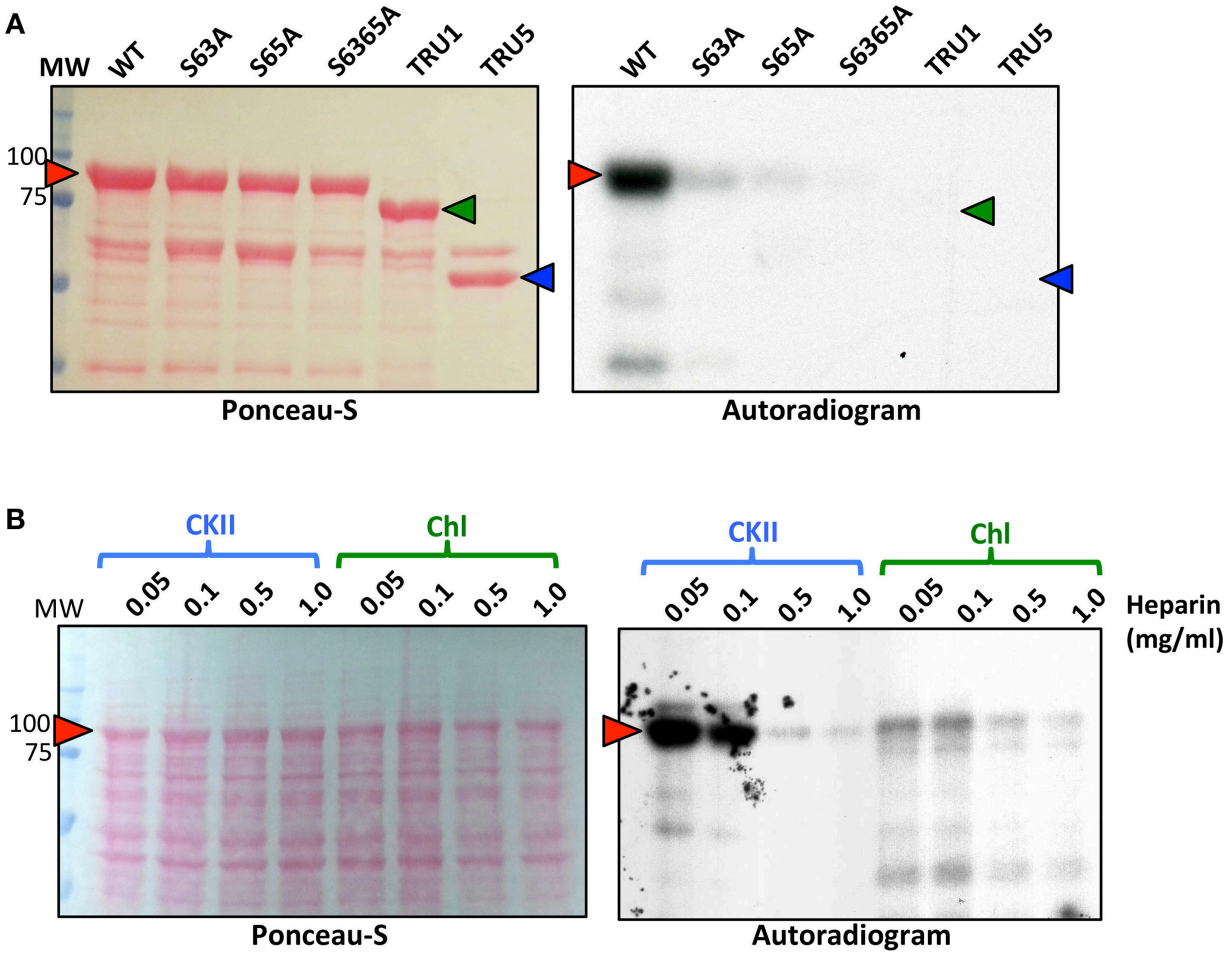
Recombinant CKII phosphorylates recombinant SS2 at S63 and S65. **(A)** S-tag immobilized, recombinant SS2 proteins containing point mutations or N-terminal truncations were radiolabeled by [γ-^32^P]-ATP using recombinant CKII. **(B)** Recombinant wild type SS2 proteins were radiolabeled using [γ-32P]-ATP in the presence of recombinant CKII or chloroplast extracts pretreated with heparin (0–1.0 mg/ml). Radiolabel led SS2 proteins (arrowheads) were separated on 10% SDS-gels, transferred to a nitrocellulose membrane and stained using Ponceau-S, ^32^P-phosphorylation was visualized using autoradiography. MW, molecular weight markers (kDa).

Recombinant SS2 proteins containing mutations at putative phosphorylation sites or successive N-terminal truncations were phosphorylated using [γ-^32^P]-ATP and chloroplast extracts as a source of protein kinases (Figure 4). When comparing the N-terminal truncations, the degree of SS2 phosphorylation was similarly reduced in S63/65A, TRU1 and TRU3. SS2 phosphorylation was further reduced in TRU4, and was completely absent from TRU5. Mutation of S63 to A decreased phosphorylation relative to wild type SS2, whereas a S65A mutation caused only a slight decrease in ^32^P-signal. The S63/65A double mutant appeared more weakly phosphorylated compared to the S63A mutation alone. SS2 serines 63 and 65 are predicted to be phosphorylated by casein kinase II (CKII), as they were followed by the CKII canonical recognition motif (SXXE/D). Recombinant SS2 was phosphorylated using recombinant casein kinase II (CKII) (Figure 5A). Again, SS2 phosphorylation decreased in both the S63A and S65A mutations, and was completely abolished by mutations that changed or removed both serine residues (S63/65A, TRU1, or TRU5). Heparin is an inhibitor of CKII in mammalian systems (Palmiter, 1973; Ghavidel et al., 1999). SS2 phosphorylation by recombinant CKII decreased with increasing heparin concentration, and was almost completely abolished by 1.0 mg/ml heparin. Similarly, phosphorylation of recombinant SS2 by chloroplast extracts was decreased with increasing heparin concentrations (Figure 5B).

### Oligomerization of SS2

**Figure 6:**
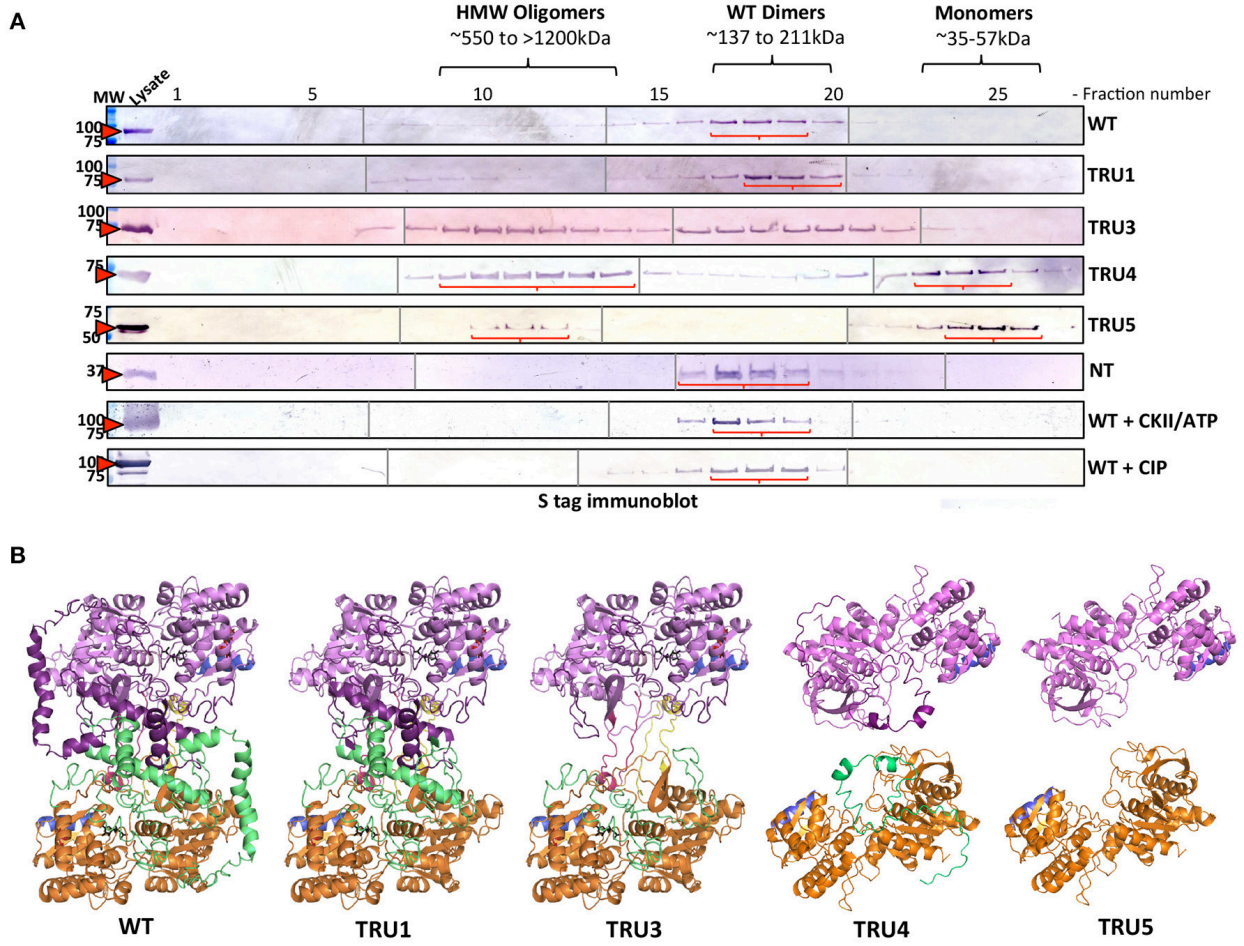
Oligomerization states of wild-type and mutated recombinant SS2 determined by permeation chromatography (GPC). **(A)** ArcticExpress bacterial pellets expressing wild type SS2 (WT) (85kDa), TRU1 (77kDa), TRU3 (70kDa), TRU4 (65kDa), TRU5 (58kDa), and Nt (25kDa), filtered and loaded to a GPC column (Superdex 20/300 GL) for protein fractionation. GPC fractions were separated on 10% SDS-gel, transferred to a nitrocellulose membrane and immunoblotted using a S-tag primary antibody. MW, molecular weight markers (kDa). **(B)** Two I-TASSER AtSS2 model 1 structures (orange/green and pink/purple) were aligned to ScGP homodimer to illustrate how AtSS2 N-terminal truncations used in **(A)** may affect homodimerization.

To investigate whether SS2 oligomerizes and which domains might be important, recombinant wild type and truncated SS2 proteins, extracted from bacterial lysate, were fractionated by GPC (Figure 6A). Wild type SS2, TRU1, and N-terminus only (Nt-only) peptides eluted in fractions corresponding to ~137–211 kDa, suggesting WT and TRU1 form dimers, whereas Nt-only could be hexameric or a higher order oligomer. TRU3 was eluted across a very broad range, equivalent to high molecular weight oligomers (>1,500 kDa) down to monomers (~71 kDa). TRU4 and TRU5 eluted in two peaks corresponding to high molecular weight oligomers (>800 kDa) and monomers (~55 kDa), respectively. To investigate the role of phosphorylation on oligomerization, wild type SS2 lysates were incubated with CKII plus ATP, or CIP. Neither phosphorylation with CKII nor dephosphorylation with CIP significantly affected SS2 elution characteristics. Structural modeling of SS2 polypeptides suggests that the N-terminal loop causes a C-terminal protrusion in I-TASSER model 1, which is less exposed as a result of N-terminal truncation in TRU4 and TRU5 (Figure 6B) and may contribute to the loss of dimerization.

### SS2 interaction with ZmSBEIIb

**Figure 7:**
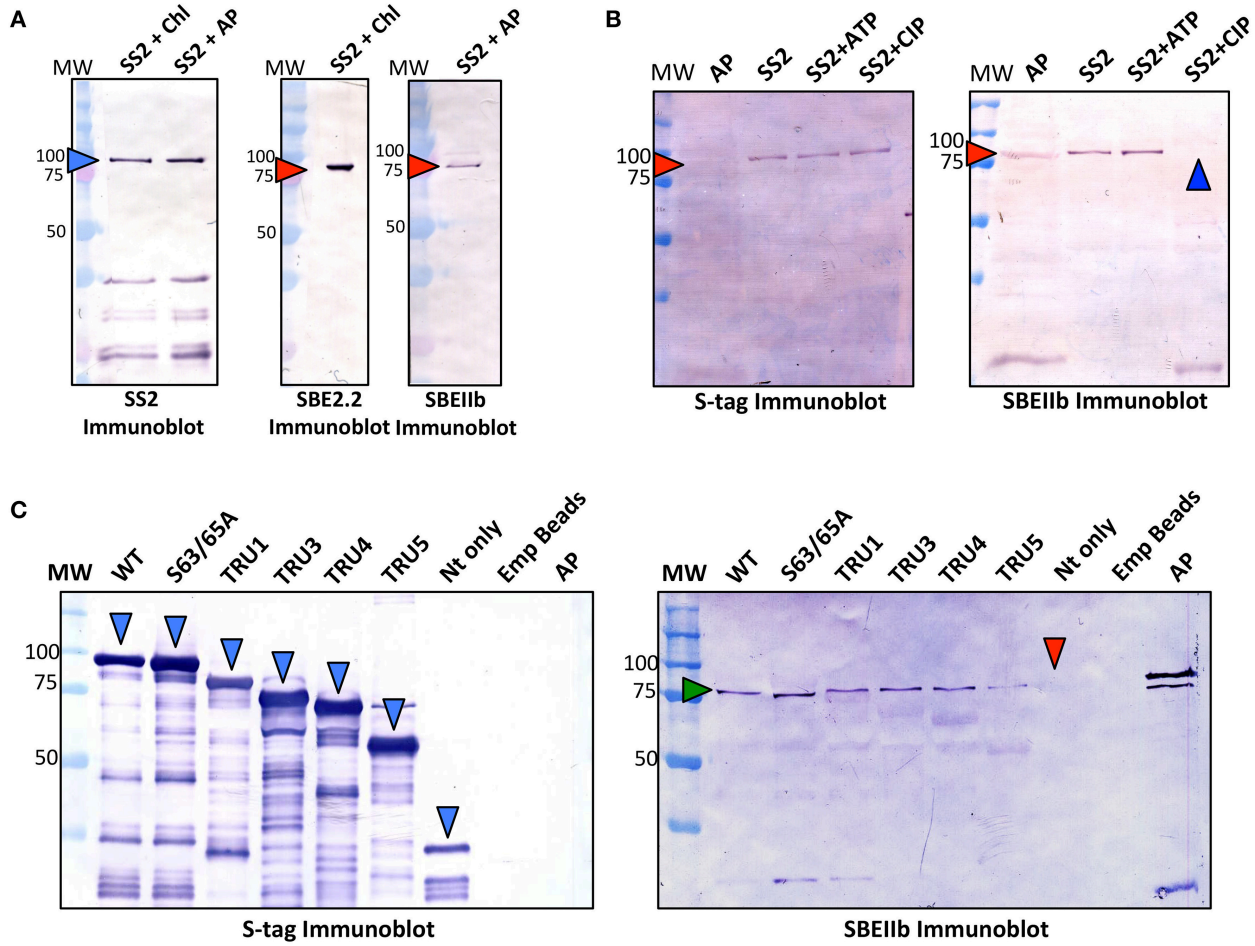
Recombinant SS2 interaction with ZmSBEIIb is phosphorylation dependent. Wild type (WT), immobilized recombinant SS2 was used as bait to reconstitute a protein complex between SS2 and SBEIIb. **(A)** S-tag immobilized SS2 was incubated with Arabidopsis chloroplast or maize amyloplast (~1 mg/ml) extracts containing 1 mM DTT, 1 mM ATP, 0.2 mM CaCl_2_ and a protease inhibitor cocktail. Reactions were incubated for 40 min, then washed to remove non-specifically bound proteins. S-tag immobilized SS2 and associated proteins were boiled in SDS-loading dye, separated on 10% SDS-gels, and immunoblotted with SS2 or SBEIIb primary antibodies. **(B)** Effects of phosphorylating/dephosphorylating conditions on SS2-SBEIIb interaction were shown by adding either 1 mM ATP or 10 U calf intestinal phosphatase (CIP) to maize amyloplast reaction mixes as described in **(A)**. **(C)** WT and mutant SS2 immobilized to S-tag beads were incubated with maize amyloplast (~1 mg/ml) extracts with 1 mM ATP, as described in **(A)**. MW, molecular weight markers (kDa).

To test whether protein complexes could be formed between SS2, and either Arabidopsis SBE2.2 or maize ZmSBEIIb, immobilized recombinant SS2 was incubated with either Arabidopsis chloroplast stroma (~1 mg/ml) or maize amyloplast stroma (~1 mg/ml), respectively in co-immunoprecipitation experiments (Figure 7A). Both AtSBE2.2 and ZmSBEIIb interacted with SS2. To further investigate the role of protein phosphorylation in affecting SS2-SBE interactions, immobilized SS2 was incubated with maize amyloplast extracts in the presence of 1 mM ATP or CIP (Figure 7B). SBEIIb bound to SS2 in untreated samples, and this interaction was enhanced by ATP, but SBEIIb did not interact with SS2 when samples were pre-incubated with CIP. The role of the SS2 N-terminus in moderating SS2-SBEIIb interactions was investigated (Figure 7C). SBEIIb interacted with wild type SS2, the S63/65A mutant, and all SS2 N-terminal truncations (TRU1, 3, 4, and 5), although the TRU5-SBEIIb interaction was reduced. In contrast, SBEIIb did not bind to the SS2 N-terminal polypeptide (NT) alone.

### Effects of phosphorylation and oligomerization on SS2 activity

**Figure 8:**
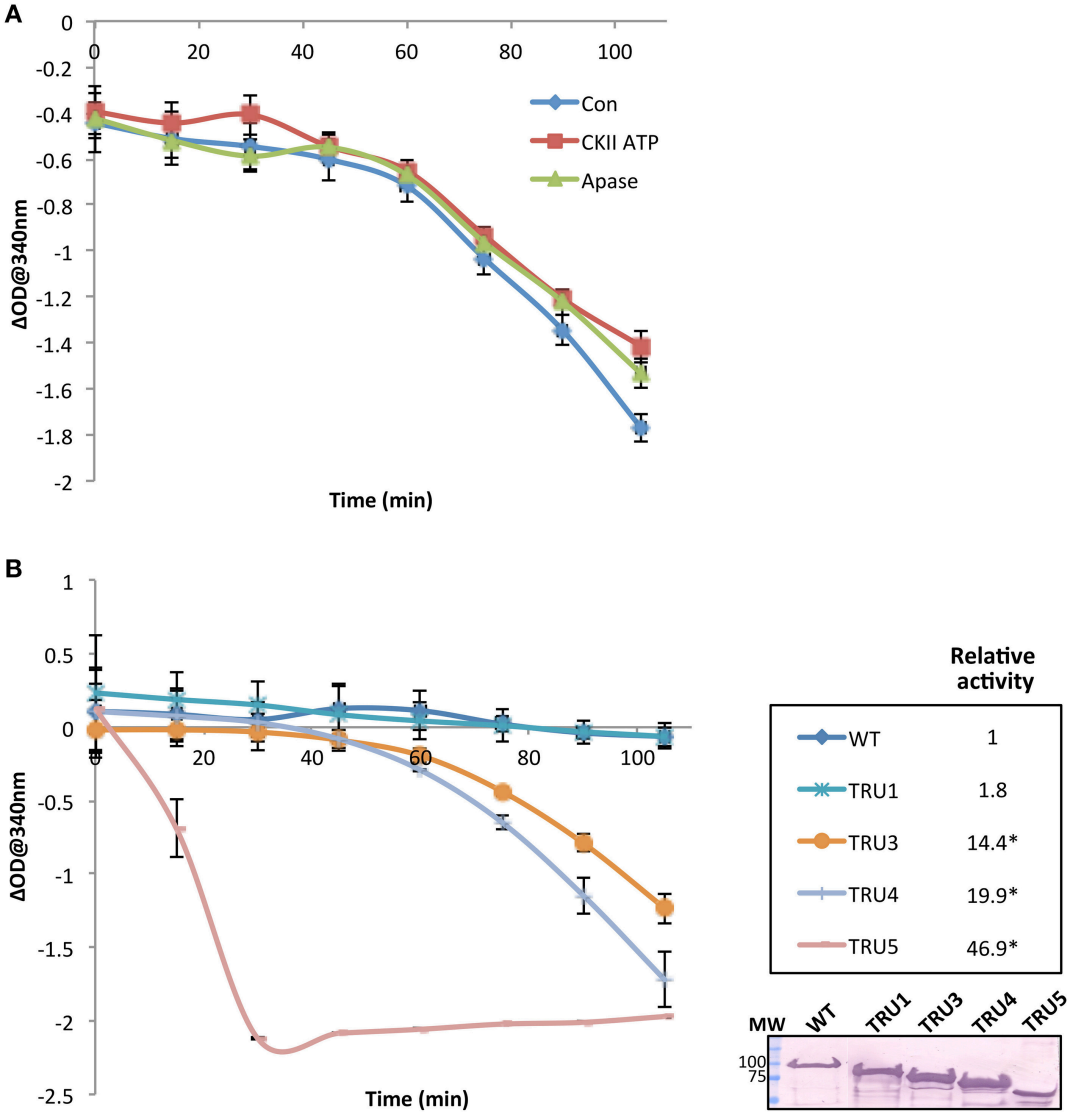
SS2 activity increases with the removal of the N-terminal region. The catalytic activity of recombinant immobilized SS2 was measured by coupling the release of ADP from ADP-glc to the oxidation of NADH. Solubilized starch was used as a glucan primer. Wild type SS2 protein was pretreated with either 1 mM ATP plus casein kinase II (CKII) or CIP (Apase) **(A)**, or measured with no pretreatment to measure the activity of wild type and mutated proteins **(B)**. Optical density at 340 nm (*n* = 3) for each treatment was deducted from a control omitting SS2 (empty beads), and linear rates determined following the 45–60 min lag phase. Significant changes (*p* < 0.05) are indicated by asterisks. Following the activity assay shown in **(B)** proteins bound to S-tag beads were boiled in SDS-loading dye, separated on 10% SDS-gels, and immunoblotted with S-tag primary antibodies. MW = molecular weight markers (kDa).

To investigate the effect of SS2 phosphorylation on catalytic activity, SS2 protein was pretreated with either 1 mM ATP plus casein kinase II (CKII) or CIP (Figure 8A). No significant changes in activity were observed under the conditions employed. Notably, there was a ~60 min lag phase prior to the onset of a linear reaction rate, the reasons for which are unclear. Catalytic activities of wild type and mutated SS2 sequences were also determined (Figure 8B). TRU1 did not exhibit significantly different catalytic activity compared to wild type. However, successive cleavage of the SS2 N-terminus in TRU3, TRU4, and TRU5 mutations significantly increased relative activity by 14-, 20-, and 47-fold that of wild type, respectively. There was again a pronounced lag phase preceding the onset of the linear reaction rate for TRU3 and TRU4, whereas the lag period disappeared completely when measuring the activity of TRU5, which was by far the most active form of the protein.

## Discussion

### AtSS2 N-terminal intrinsic disorder

The purpose of this study was to identify potential mechanisms by which Arabidopsis SS2 might be regulated post-translationally. When comparing the AtSS2 sequence to other SS2 orthologs, the N-terminal region exhibited no significant conservation, while the central and C-terminal domains were highly conserved across all species (Figure 1). The C-terminal conservation relates to the shared function of SS2 orthologs, as the central catalytic region contains the characteristic ADP-glc binding motif (KTGGL) and catalytic cleft, which is generally conserved amongst glycogen/starch synthases and phosphorylases (Mahrenholz et al., 1988; Furukawa et al., 1990, 1993; Nichols et al., 2000; Sheng et al., 2009). Structural predictions of the variable SS2 N-terminus indicated that it could be intrinsically disordered (Figure 2). Intrinsically disordered regions retain no stable conformation but, instead, dynamically transition between multiple conformations. *In vitro* evidence suggested that the SS2 N-terminus may be intrinsically disordered. Firstly, when separating the N-terminus (Nt) of SS2 by GPC, it eluted in fractions corresponding to relatively large oligomers containing at least 6-8 subunits (Figure 6) When expressed recombinantly, intrinsically disordered proteins (IDP) are prone to aggregation (Lebendiker and Danieli, 2014). When separated on SDS-PAGE, IDP/IDR's typically migrate as though they exhibit a higher molecular mass (Calçada et al., 2015). This abnormal SDS-PAGE migration pattern was seen for the wild type SS2 proteins, which has a predicted molecular weight of 85 kDa, but migrated close to the 100 kDa marker (Figures 4–6), and has previously been observed for the SSIIa isoform in wheat endosperm (Li et al., 1999).

### SS2 phosphorylation by chloroplast protein kinases (CKII)

The occurrence of IDR's is often correlated with post-translational modification, as the inherent structural plasticity facilitates protein-protein interactions and/or protein phosphorylation (Wright and Dyson, 2015; Niklas et al., 2018). Within the putative AtSS2 N-terminal IDR, two protein phosphorylation sites (S63/65) were previously identified by high-throughput mass spectrometry of Arabidopsis whole leaf extracts (Reiland et al., 2009). Mutation of either S63A or S65A decreased SS2 phosphorylation relative to wild type, and phosphorylation was completely abolished by removing the entire N-terminus (TRU5) (Figure 4). Some residual ^32^P-signal was detected following mutation of both S63 and S65, suggesting other possible SS2 phosphorylation sites. Alternative phosphorylatable residues are likely confined to the N-terminus, as removing the N-terminus completely abolished SS2 phosphorylation, though none were identified in this study.

Analysis of the region surrounding S63/65 identified the +1 to +3 residues as being highly acidic (DDD), which is consistent with the canonical binding motif of casein kinase II (CKII) (Meggio et al., 1994). Chloroplast-localized CKII activity regulates many developmental pathways in Arabidopsis, including the coordination of photosynthesis and light sensing, both by phosphorylating enzymes involved in photosynthesis and their transcriptional regulators (Schönberg and Baginsky, 2012). In this study, recombinant CKII strongly phosphorylated the wild type SS2 protein at S63 and S65, and mutations removing these targets, including N-terminal truncations (TRU1 and TRU5), completely abolished SS2 phosphorylation (Figure 5A). To investigate whether a CKII-like kinase might be responsible for SS2 phosphorylation in Arabidopsis chloroplasts, a CKII-specific inhibitor, heparin, was used to pretreat recombinant CKII and chloroplast extracts prior to recombinant SS2 phosphorylation. Phosphorylation of SS2 by chloroplast extracts decreased with increasing concentrations of heparin (Figures 5B,C), suggesting that a chloroplast-localized CKII is responsible for SS2 phosphorylation. Recombinant CKII has been shown to phosphorylate starch branching enzyme 2.1 (SBE2.1) and fibrillin, the latter of which interacts with SS4 and enables its association with plastoglobuli (Gámez-Arjona et al., 2014; Schönberg et al., 2014). As chloroplastic CKII has been shown previously to phosphorylate other starch metabolism-associated enzymes and has broad substrate specificity, it may well have a broader role in regulating and coordinating starch biosynthesis, although the function of SS2 phosphorylation remains unclear.

### Oligomerization of recombinant SS2

Size fractionation (GPC) of recombinant wild type SS2 (85 kDa) and TRU1 (77 kDa) indicated that they likely exist as homodimers (~211–137 kDa) (Figure 6). Successive removal of the SS2 N-terminus resulted in elution of TRU4 (65 kDa) and TRU5 (58 kDa) as either high molecular weight oligomers (>800 kDa) or monomers (~55 kDa). TRU3 (70 kDa) eluted in very broad “peaks,” suggesting multiple oligomeric states ranging from high molecular weight (>1,200 kda) to monomers (~71 kDa). Size fractionation of the N-terminal region alone (Nt) suggested it exists in larger oligomeric complexes involving at least six subunits (25 kDa/subunit). Taken together, GPC data indicate that full-length, mature SS2 forms a homodimer that is dependent on the presence of the N-terminus, but also suggests that the N-terminus alone cannot form stable homodimers by itself.

Currently, there is no solved crystal structure for a SS2 isoform in any plant species. This could be due to the putative intrinsic disorder of the SS2 N-terminus, which may make efforts to produce useable SS2 crystallography data more difficult. However, there are structures available for barley SS1 (PDB ID: 4HLN) and rice GBSS (PBD ID: 3VUF), the former of which was used as a template to predict a homology model of SS2. In the predicted model of SS2 (I-TASSER AtSS2 model 1), a portion of the SS2 N-terminal region was threaded through the SS2 central catalytic domain and caused a loop protrusion (Figure 3). Comparing this predicted structural model to structural analogs (e.g., ScGP, PDB ID: 1YGP), revealed that this central loop protrusion may lead to interaction between subunits within a homodimer, and that this interaction may be stabilized by interactions between the N-termini of each monomer. The GPC elution patterns of wild type and the N-terminally-truncated SS2 proteins support this model. As shown in (Figure 6B), in wild-type SS2, TRU1 and TRU3, the N-terminal loop remains threaded through the central catalytic structure, but is removed in TRU4 and TRU5. If this SS2 loop protrusion is required for homodimerization, TRU4 and TRU5 proteins would be unable to form the dimer observed for the wild-type sequence, which is reflected in their GPC elution pattern as monomers. Further, N-terminal interactions may also be required for SS2 homodimer stabilization. The necessity of N-terminal interactions for homodimer formation is reflected in the TRU3 GPC elution pattern. In the case of TRU3, the N-terminus would still be capable of causing the central loop protrusion, but the majority of the N-terminus is removed. If N-termini interact directly within the homodimer, TRU3 may not be able to form a stable dimer, causing multiple conformations from high molecular weight aggregates to dimers to monomers. The inability of the expressed SS2 N-terminus to form homodimers indicates that there is some component of the C-terminus (e.g., the central loop protrusion) that is required for stable homodimerization, which further supports this model.

The predicted structural ortholog to AtSS2, ScGP, requires protein dephosphorylation to stabilize homodimerization (Figure 3), a mechanism which is conserved amongst eukaryotic glycogen synthases and phosphorylases (Lin et al., 1996; Baskaran et al., 2010). However, protein phosphorylation did not affect its ability to homodimerize, as SS2 treated with either CKII/ATP or CIP showed similar GPC elution patterns to untreated SS2 (~211–137 kDa). Plant starch synthases are evolutionarily related to bacterial glycogen synthases, as SS's are thought to have originated from gene duplication of cytosolic SS's in early *Archaeplastidia*, and by lateral gene transfer from an intracellular pathogen (*Chlamydiae*) (Huang and Gogarten, 2007; Deschamps et al., 2008; Moustafa et al., 2008). Unlike eukaryotic GS/GP's, bacterial GS's form homodimeric complexes independent of allosteric regulators and post-translational modification (Furukawa et al., 1994; Buschiazzo et al., 2004). Eukaryotic GS's and plant SS's likely share a common ancestor in bacterial GS's, from which eukaryotic GS's diverged and developed novel regulatory mechanisms and substrate specificities [e.g., UDP-glc vs. ADP-glc, respectively (Preiss and Romeo, 1990; Ball et al., 2011)]. It is possible that although AtSS2 shares structural similarity to eukaryotic GS/GP's, its regulation may be more similar to its bacterial GS ancestors.

### Heteromeric enzyme complexes between SS2 and SBE

Previous studies of maize, wheat, rice, and barley endosperm have indicated that SSIIa interacts with SBE-II class enzymes and that this process is ATP-dependent (Tetlow et al., 2004b, 2008; Hennen-Bierwagen et al., 2008; Ahmed et al., 2015; Crofts et al., 2015). Recombinant S-tag immobilized SS2 was used as bait to test whether Arabidopsis chloroplast SBE2.2 or maize amyloplast SBEIIb could reconstitute a similar SS2-SBE complex, as prior to this study, such complexes have not been reported in leaves of dicotyledonous species (Figure 7A). Recombinant AtSS2 interacted with both AtSBE2.2 and ZmSBEIIb, and this interaction was lost in the presence of a non-specific phosphatase (CIP) (Figure 7B). SBEIIb interacted with all SS2 N-terminal truncations and the S63/65A mutant, but not with the N-terminus (Nt) only (Figure 7C). As SS2 protein phosphorylation was shown in previous experiments to occur exclusively in the N-terminus (Figure 5), the interaction between TRU5 and SBEIIb indicates that phosphorylation of SS2 is not required for complex assembly. Rather, the phosphorylation of SBEIIb or a yet-undetermined protein may underpin the ATP-dependence of SS2-SBEIIb HEC assembly, which will be discussed further below. The lack of direct interaction with the Nt peptide suggests that the SS2-SBEIIb interaction interface is not located in this region and that the N-terminus is not required for this interaction. However, it is possible that the N-terminus may contribute in some way to HEC assembly, as the interaction between TRU5 and SBEIIb was reduced relative to WT. Further, the observation that TRU4 and TRU5 are monomeric (Figure 6) suggests that SS2 homodimerization is not required for SS2-SBE complex assembly.

Thus, although ATP appears to be required for SS2-SBE interaction, SS2-specific phosphorylation is not required. Maize SBEIIb, which is orthologous to Arabidopsis SBE2.2, has been shown to be phosphorylated at three residues (S286, S297, and S649), although the role of these sites in regulating interactions with SSIIa has yet to be determined (Makhmoudova et al., 2014). Two of these phosphoresidues are conserved in Arabidopsis SBE2.2 (S290/S301), and it is possible that their phosphorylation plays a role in mediating HEC assembly with SS2. Alternatively, the phosphorylation of other plastidial regulators may be required. For example, 14-3-3 proteins, which often interact in a phosphorylation-dependent manner with their client targets, have been shown to regulate other enzymes of carbon metabolism [such as sucrose phosphate synthase (Huber and Huber, 1992; Huber et al., 2002)]. 14-3-3's have also been shown to be associated with starch granules in Arabidopsis leaves and maize endosperm (Sehnke et al., 2001). Thus, the ATP-dependence of SS2-SBE2.2 complex assembly may be due to associations with other stromal enzymes, possibly in combination with SBE2.2 phosphorylation, *in vivo*.

### Regulation of SS2 catalytic activity

**Figure 9:**
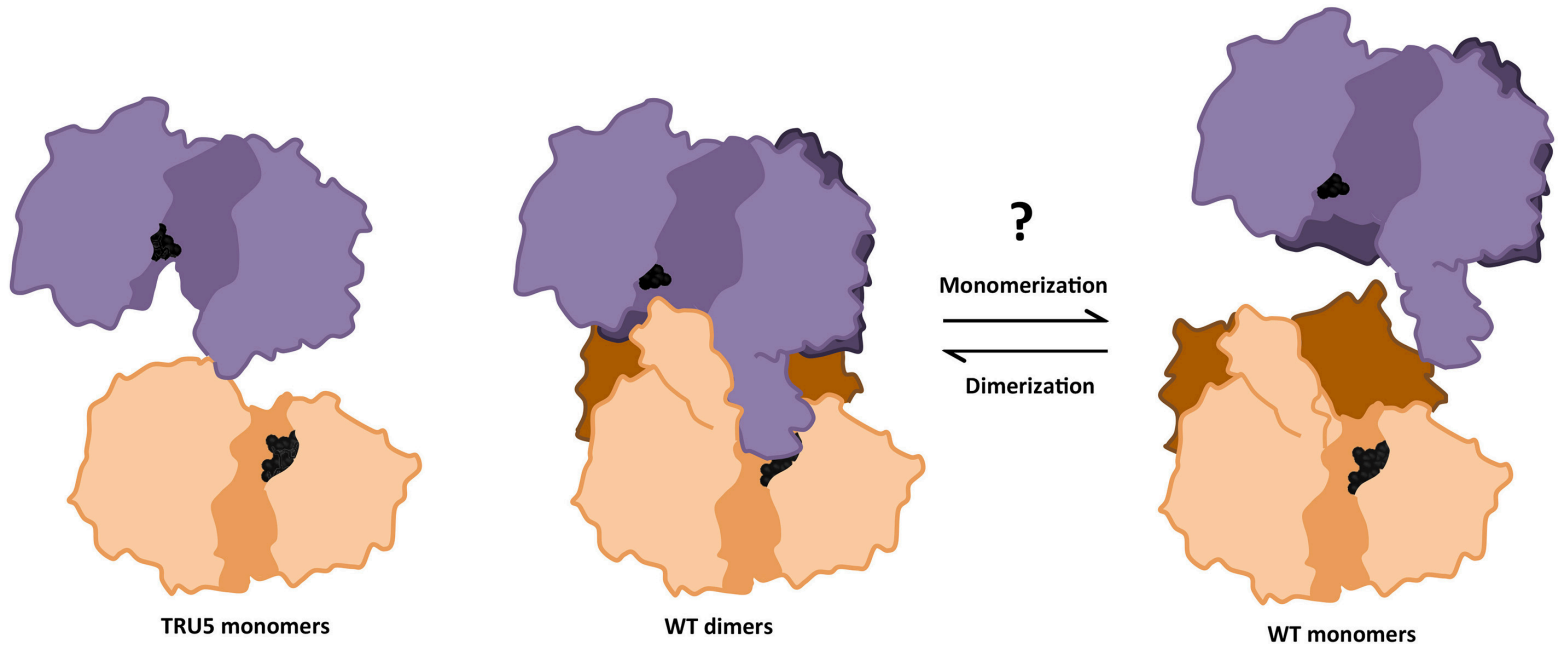
Transition between monomeric and dimeric conformations may regulate SS2 catalytic activity. ITASSER AtSS2 model 1 aligned using dimeric ScGP (PDB ID: 1YGP) and SS2 SWISS model (omitting the SS2 N-terminal region) were used to illustrate the effects of wild type (WT) oligomerization and TRU5 truncation, respectively, on substrate accessibility (ADP-glc, black spheres) in the catalytic cleft (shaded region). Omitting the N-terminal regions (dark purple/orange) prevents the central loop extension and may facilitate access for substrates.

Investigation of the role of the N-terminal region and, by extension, homodimerization, revealed a marked increase in SS2 activity with each successive cleavage of the SS2 N-terminus, suggesting that the latter may regulate/inhibit SS2 activity (Figure 8B). Full-length SS2 is dimeric, but removal of the entire N-terminal region (TRU5) results in SS2 monomerization as well as high molecular weight oligomerization (Figure 6). The central loop protrusion (Figure 9), which is putatively caused by the presence of the N-terminus, can be postulated to prevent the binding of substrates [ADP-glc or α-(1,4) glucans] in the catalytic cleft. Increasing truncation of the N-terminus may allow the catalytic cleft to become more accessible to substrates. Whether the large increase in catalytic activity is due to the formation of monomers or higher order oligomers of SS2 is unclear. Previous truncation studies with recombinant SSI and SSII isoforms from developing maize endosperm suggest that the N-terminus is not essential for the catalytic activity of either cereal SS isoform, although no attempt was made to determine the aggregation state of the purified recombinant proteins (Imparl-Radosevich et al., 1998, 1999). This may suggest a difference in the role of the N-terminus in regulating activity of the leaf enzyme of Arabidopsis, where starch turns over diurnally, versus regulation of SSII in cereal endosperm which is involved in the accumulation of storage starch to support seed germination.

Transitions between dimeric and monomeric conformations could regulate SS2 activity similarly to eukaryotic GP/GS's, with which SS2 is predicted to share structural similarity (Baskaran et al., 2010; Palm et al., 2013). Oligomeric enzymes can undergo lag phases as a result of conformation transitions (Tipton, 2002), as was apparent in the activities of full-length SS2, TRU1, TRU3, and TRU4 (Figures 8A,B). By some unknown mechanism, an equilibrium between oligomeric/dimeric/monomeric conformations may contribute to this lag phase. Consistent with this, no lag phase in SS2 activity was observed in TRU5, which exists predominantly as a monomer (Figure 6), supporting the hypothesis that oligomerization could regulate SS2 catalysis. This raises the question of how this transition is regulated, as neither SS2 homodimerization or catalytic activity are affected by phosphorylation, unlike ScGS/GP (Figures 6, 8A). It is possible that other, yet unidentified post-translational regulatory mechanisms may initiate this transition, such as redox modulation. Redox modulation has been shown to regulate the activities of other starch metabolism enzymes (Glaring et al., 2012). Thus, regulation of stromal SS2 activity by alterations in its oligomeric state, the mechanisms that underpin this phenomenon, and its implications for its interactions with other enzymes of starch biosynthesis require further investigation.

## Conclusion

**Figure 10:**
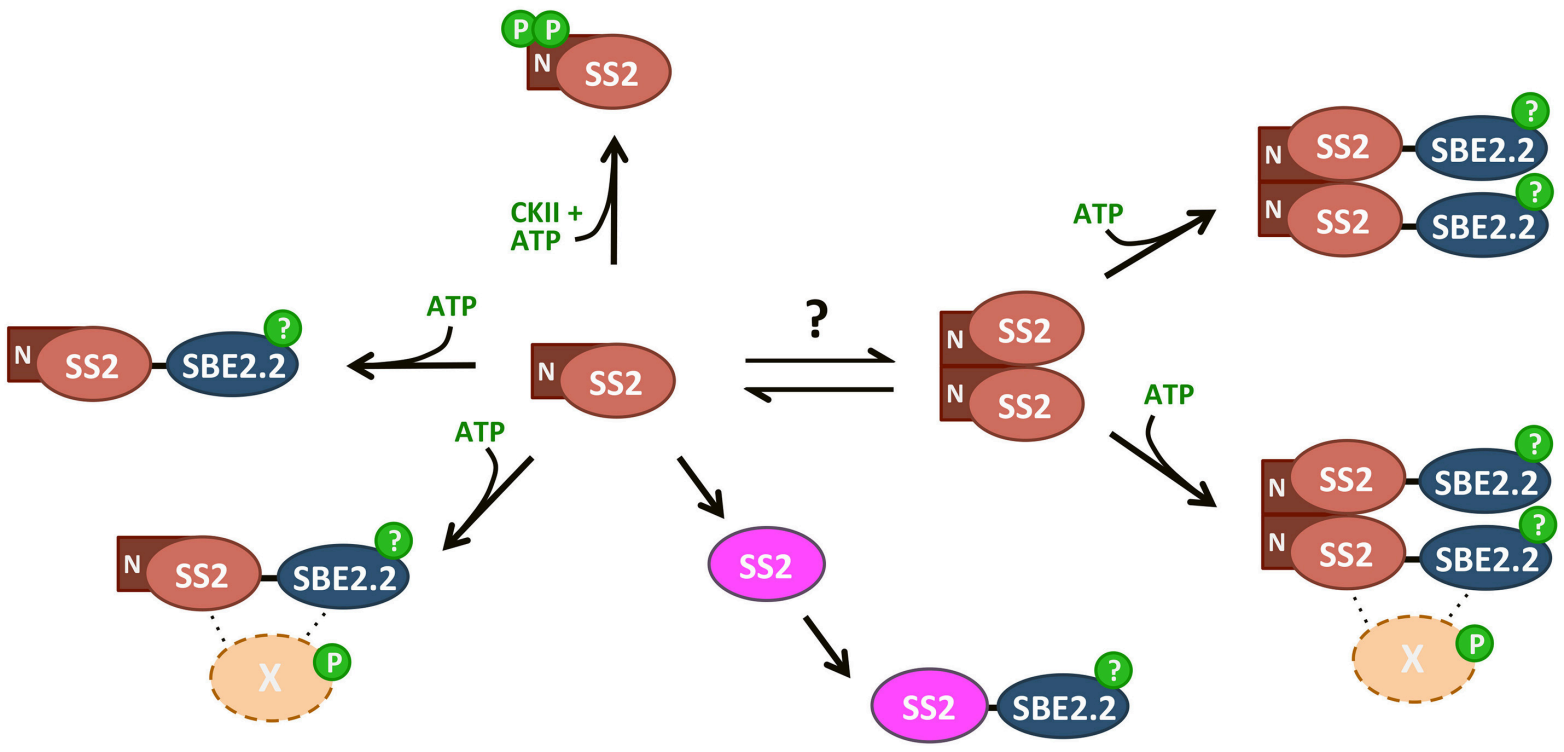
Summary of SS2 post-translational modifications. SS2 can form homodimers dependent on the N-terminal region, removal of which results in monomerization. An equilibrium between monomeric and dimeric states could contribute to the regulation of catalytic activity, as TRU5 monomers are much more active than wild type dimers. Both monomeric and dimeric SS2 can interact with SBE in an ATP-dependent manner, but SS2 phosphorylation is not required. Phosphorylation of SBE2.2 or an as-yet unknown protein (X) may facilitate this interaction. SS2 can be phosphorylated at two N-terminal serines (S63/65), possibly by a chloroplast casein kinase II.

In summary, wild type SS2 was shown to form homodimers, dependent on its intrinsically disordered N-terminus. Dimerization was unaffected by SS2 phosphorylation state, confirming that SS2 regulation may be more similar to its bacterial GS ancestor than to eukaryotic GS's. Monomerization seems to enhance SS2 activity, but does not affect its ability to interact with SBE. Whether SS2 undergoes transitions between oligomeric states similar to eukaryotic GS/GP's as a regulatory mechanism *in vivo* remains unknown. SS2 was shown to be phosphorylated at S63/65, which may be phosphorylated *in vivo* by a chloroplast CKII. SS2 phosphorylation state appears not to regulate its catalytic activity or protein-protein interaction with SBE2.2, thus, the role of S63/65 phosphorylation remains unclear. It is possible that SS2 phosphorylation regulates interactions with other, unknown proteins and/or its localization within the stroma or granule of the chloroplast. A model outlining the possible mechanisms by which SS2 is regulated highlights the key results present in this work (Figure 10). Future investigations into the physiological relevance of SS2 regulation, *in planta*, should elucidate the role(s) of SS2 phosphorylation and the mechanisms regulating its oligomeric state in the plastid stroma.

## Author contributions

ME and IT conceived the study and secured funding to support research activities. ME, IT, and JP designed all experiments and analyzed the data. JP conducted all experiments, collected data, and wrote the manuscript. ME, IT, and JP edited the manuscript. All authors read and approved the manuscript.

### Conflict of interest statement

The authors declare that the research was conducted in the absence of any commercial or financial relationships that could be construed as a potential conflict of interest.
